# Simultaneous Grafting Polymerization of Acrylic Acid and Silver Aggregates Formation by Direct Reduction Using γ Radiation onto Silicone Surface and Their Antimicrobial Activity and Biocompatibility

**DOI:** 10.3390/molecules26102859

**Published:** 2021-05-12

**Authors:** Marlene A. Velazco-Medel, Luis A. Camacho-Cruz, Héctor Magaña, Kenia Palomino, Emilio Bucio

**Affiliations:** 1 Departamento de Química de Radiaciones y Radioquímica, Instituto de Ciencias Nucleares, Universidad Nacional Autónoma de México, Circuito Exterior, Ciudad 7 Universitaria, Ciudad de México 04510, Mexico; c-camacho-la@comunidad.unam.mx; 2 Faculty of Chemical Sciences and Engineering, Autonomous University of Baja California, University Boulevard No. 14418, Otay Mesa, Tijuana 22390, Mexico; hector.magana@uabc.edu.mx (H.M.); kenia.palomino@uabc.edu.mx (K.P.)

**Keywords:** silicone, grafting, silver nanoparticles, radiation, antimicrobial, cytocompatibility

## Abstract

The modification of medical devices is an area that has attracted a lot of attention in recent years; particularly, those developments which search to modify existing devices to render them antimicrobial. Most of these modifications involve at least two stages (modification of the base material with a polymer graft and immobilization of an antimicrobial agent) which are both time-consuming and complicate synthetic procedures; therefore, as an improvement, this project sought to produce antimicrobial silicone (PDMS) in a single step. Using gamma radiation as both an energy source for polymerization initiation and as a source of reducing agents in solution, PDMS was simultaneously grafted with acrylic acid and ethylene glycol dimethacrylate (AAc:EGDMA) while producing antimicrobial silver nanoparticles (AgNPs) onto the surface of the material. To obtain reproducible materials, experimental variables such as the effect of the dose, the intensity of radiation, and the concentration of the silver salt were evaluated, finding the optimal reaction conditions to obtain materials with valuable properties. The characterization of the material was performed using electronic microscopy and spectroscopic techniques such as ^13^C-CPMAS-SS-NMR and FTIR. Finally, these materials demonstrated good antimicrobial activity against *S. aureus* while retaining good cell viabilities (above 90%) for fibroblasts BALB/3T3.

## 1. Introduction

Silicone elastomers (a.k.a. silicone rubbers, silicones, polydimethylsiloxane, PDMS) are polymers which possess properties that are very important for the development of devices on medicine [1,2,3]. For instance, these materials are biocompatible, readily accessible, and easy to manipulate. Despite these advantages, PDMS by itself is prone to microbial contamination through the formation of pathogenic biofilms on its surface [4,5], triggering infections in patients who may already be compromised [6]. This stark disadvantage can be mitigated by the modification of PDMS via grafting, strategy which has already been used extensively to improve the properties of many materials by modifying the characteristics of materials with behaviors such as biocompatibility, hydrophilicity, conductivity, and antimicrobial activities [1,2,3].

One of the most common strategies to provide antimicrobial activity to surfaces (e.g., carbon nanomaterials [7,8], inorganic substrates [9,10], and polymeric substrates [11]) is the immobilization of antimicrobial agents through interactions between the antimicrobials and grafted polymer chains onto the base material or by covalent attachment of the antimicrobial to the surface of the base material [12,13]. These antimicrobial agents may be both organic (classical molecular antibiotics) or inorganic (metallic particles) in nature; however, the latter have attracted more attention because the use of classical antibiotics still increase concerns for bacterial antibiotic resistance [14,15]. Most inorganic antimicrobial compounds are metal salts or metal nanoparticles, such as those containing copper, titanium, zinc, and silver, whose biggest advantage is the fact that they can not only be used by themselves, but they can also be combined onto organic substrates such as polymers, or even work alongside other antimicrobials on complicated substrates. For example, Pissinis et al. (2018) performed the functionalization of Ti/TiO_2_ surfaces with a mixture of ampicillin and silver nanoparticles (AgNPs) to stop the propagation of Gram-positive *S. aureus* [16]. This is one of many examples employing metals as antibacterial substances.

One system which has shown great effectiveness as an antibacterial substrate is silver. Silver ions, colloidal silver, and AgNPs have demonstrated cytotoxicity against several pathogens because when this metal interacts with the nitrogenous bases of nucleic acids, it produces oxidative stress in cells [17,18,19,20]. Because of this, silver salts have already been encapsulated in medical devices to avoid infections, such as catheters or wound dresses. AgNPs have also been immobilized in silicone and other medical-grade polymers [21].

Several strategies for the production of AgNPs onto polymer substrates have been reported [22,23,24,25,26]. For example, the synthesis of AgNPs in-between polyacrylic acid grafts has already been achieved by chemical reduction of Ag^+^ to form hybrid materials [27]. In this example, it is worthy to note that polyacrylic acid grafts not only support the silver particles, but also provide stabilization comparable to citrate or alginate ions in other systems [28,29]. The immobilization of AgNPs onto PDMS has also been thoroughly studied by different deposition techniques. The most conventional pathways are by the diffusion of AgNO_3_ inside the elastomer and subsequent interaction with a reducing agent, such as NaBH_4_ or sodium citrate [30,31], the diffusion of preformed nanoparticles inside a polymer, or the electrodeposition onto the polymeric surface [32]. For example, López-Saucedo et al., 2018 immobilized preformed AgNPs onto medical grade polytetrafluorethylene to avoid bacterial adhesion [33] and Pazos-Ortiz et al., 2017 used polycaprolactone fibers to retain AgNPs [34]. Additional techniques have been used to attach or immobilize these particles to silicone elastomers, for example, the synthesis of composites of PDMS + AgNPs [35] or the presence of polymers or stabilizers in the process [36].

Although these strategies are both effective to produce antimicrobial substrates using silver, practically all these implementations need at least two synthetic steps; namely, the grafting of a functional molecule or polymer able to support the nanoparticles, the immobilization of silver as nanoparticles or ions, and, in case of first immobilizing silver ions, their reduction to produce silver nanoparticles. Therefore, the search of simplified synthetic pathways is an important goal. A solution to this dilemma would be the simultaneous grafting of polymeric chains, and the direct reduction of silver ions in the reaction media. To achieve this goal, we considered the possibility of using high-energy radiation as a pathway to polymerization initiation and direct reduction of silver ions.

Several stimuli-responsive polymers have been grafted onto different polymer matrixes using radiation [37,38]; for example, acrylic acid has been grafted onto silicone using gamma radiation, laser emission, and plasma. Cabana et al., 2017 achieved the retention of gold nanoparticles to add antimicrobial properties [39]. Alongside this, direct reduction of silver ions to AgNPs using high-energy radiation is possible in aqueous or polar media because when radiation interacts with these solvents, reductive species such as hydrogen radicals (H^·^) and solvated electrons (e^−^_aq_) are readily formed [40,41,42,43,44,45].

Previous reports on this topic mainly focus on the study of the effects of the radiation dose, radiation intensity, and salt concentration on the morphology and size of the synthesized. With these studies it has been discovered that the formation of these metallic nanoparticles is highly dependent on synthesis time and irradiation dose [41,42,46]. The most remarkable aspect to note is the effect of the dose in the shape of the particle; for instance, Abedini et al. (2016) reported that reactions conducted at doses of 30 kGy promote the formation of spherical particles. However, at higher doses, nanoplates or triangular particles are formed [41]. Despite this, changes in morphology do not affect AgNPs’ antimicrobial effects [47].

Profiting from the benefits of AgNPs addition to polymer matrices, this project proposes the use of ionizing radiation (coming from a ^60^Co radioactive source) to perform the simultaneous reduction of Ag^+^ and polymerization of AAc and EDGMA for the in-situ immobilization of AgNPs. By altering variables during the synthetic procedure such as concentration of the monomers, concentration of the silver ions, and irradiation dose, different materials were obtained and characterized with techniques such as SEM microscopy with EDX elemental analysis, FTIR, and ^13^C-CPMAS-SS-NMR. Subsequent antimicrobial and biocompatibility tests were performed to test the applicability of these novel materials.

## 2. Results and Discussion

### 2.1. Grafting Reaction and Silver Particle Formation Assisted by Gamma Radiation

As we previously reported [48], the choice of solvent is crucial to obtain bulk or surface grafts in a polymer matrix. For metal immobilization, it is necessary to obtain polymer chains on the base surface to favor the nanoparticle formation onto the surface rather than inside the bulk. Silicone films (SF) are highly hydrophobic; therefore, polar solvents do not interact with PDMS, thus the choice of the solvent should be carried out so that there is low (but not null) diffusion inside the matrix. To select the solvent, it is then necessary to reference previous studies on the swelling of PDMS on different solvents [49]. The grafting reaction was carried out through this analysis in a solvent mixture (EtOH:H_2_O). A low concentration of the mixture of monomers (20% *v*/*v*) was used to prevent solubility issues with the monomers, and monomer molar ratios of 3:1 and 5:1 (AAc:EGDMA) were tested on preliminary experiments.

**Figure 1:**
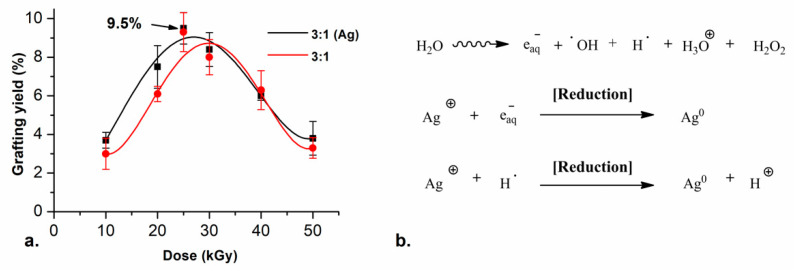
(**a**) Grafting yield of (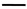) SF-*g*-(AAc-*co*-EGDMA) + Ag and (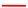) SF-*g*-(AAc-*co*-EGDMA) in function of the dose with monomer concentration fixed at 20% (*v*/*v*) and (**b**) Irradiation-reduction mechanism of silver reduction.

The effect of the irradiation dose was tested by irradiating the samples at different doses with an intensity of ~10 kGy h^−1^. The highest grafting yield was obtained with an irradiation dose of 25 kGy. Higher doses did not further increase the yield; however, the reactions did not proceed at these doses. This may be due to rapid termination rates at these higher irradiation doses. Grafting yields (GY) of about 10% were obtained by irradiating the monomer solution and the SF simultaneously, using the 3:1 monomer ratio (red line in Figure 1a). For the 5:1 ratio, the grafting reaction did not proceed because the formation of the unbounded polymer is preferred, the highest GY with this condition was 2.5%.

For the systems containing silver, the same synthetic conditions used before were evaluated, but adding different concentrations of AgNO_3_ to the reaction media. With all tested conditions, the grafting reaction and the reduction of the silver ions were reached successfully in one step. Furthermore, the presence of the silver nitrate (5 mM) did not change the GY on the films. The highest GY with these conditions was 9.5%, which was equivalent to the grafting yield corresponding to the reaction without the silver salt (black line in Figure 1a). The most notable change in the films was that they turned colorful, from clear to yellow and orange, similar to other reported elastomer + AgNPs [35].

The reduction reaction of silver ions was carried out by an irradiation–reduction mechanism assisted by gamma radiation. The radiolysis of the water promotes different reactive species (Figure 1b), which react with the silver salt and trigger a redox reaction.

The atom agglomeration drives the growth of small spherical and quasi-spherical particles in the nanoscale. The acrylic acid moieties of the monomer decrease the pH of the reaction medium to 5, thus the hydrogen radical is the main reducing agent for silver in these conditions. Additionally, the polycarboxylic acids in the polymer graft work as stabilizers in the reactions which allow the retention of the AgNPs between the chains in the graft.

### 2.2. Characterization of the Films

**Figure 2:**
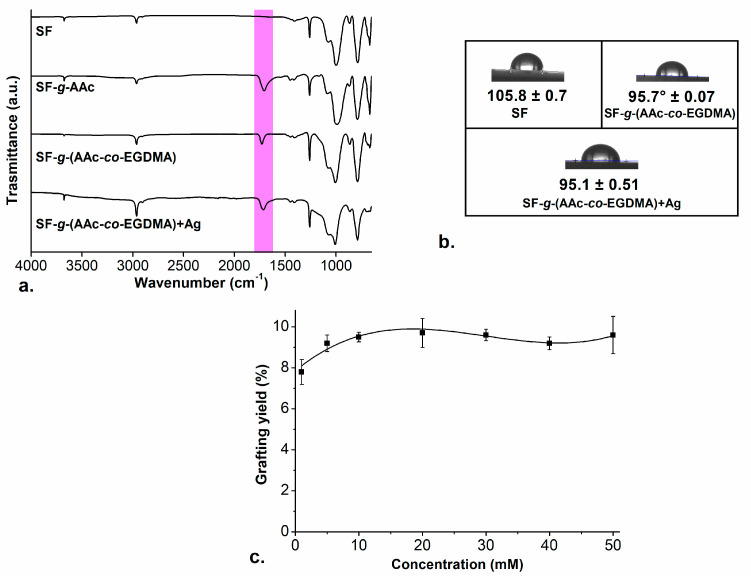
(**a**) FTIR-ATR spectrum for the modified films; (**b**) contact angle images of the films; and (**c**) grafting yields of (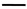) SF-*g*-(AAc-*co*-EGDMA) + Ag in function of the concentration of AgNO_3_.

FTIR-ATR spectra (Figure 2a) show changes regarding the initial SF but not between the film SF-*g*-(AAc-*co*-EGDMA) compared with the SF-*g*-(AAc-*co*-EGDMA) + Ag. According to these spectra, we can conclude that there is polymer graft present on both surfaces after the radiation, even when there is silver in the system, there are no changes in the absorption bands which correspond to the typical functional groups reported for silicone modified with AAc [50,51].

The spectrum of pristine SF showed strong bands corresponding to the Si-O-Si stretching and Si-CH_3_ bending vibrations at ~1000 cm^−1^ and ~750 cm^−1^, respectively (Figure 1b, SF). The short band at ~2900 cm^−1^ is attributed to the typical band for C-H stretching, present in all the spectra. The absorption band at ~1710 cm^−1^ is a characteristic band for the C=O stretching confirming the presence of this group on the functionalized versions of SF.

Aac:EGDMA grafting onto SF was confirmed further with equilibrium water content (EWC%) tests. In these tests, SF-*g*-(AAc-*co*-EGDMA) with GY~9.5% demonstrated swelling properties in deionized water (DI water) and phosphate buffer saline (PBS) solution (in contrast to pure SF); this fact aided to confirm the AAc grafts onto the SF. The EWC% for the films in DI water and PBS was ~3.7 (24 h) and 8.8% (30 h), respectively (for graphs, see Supplementary Material). Although DI water and PBS both have a pH above the pKa of AAc (pKa 4.7), the dissolved salts in the PBS change the ionic strength in the solution, which trigger a variation in the osmotic pressure inside and outside of the graft. This change conduces to the ion flow from the solution to the free volume between polymeric chains, this effect is similar as other hydrogel swelling behaviors [52].

Additional to EWC% studies, contact angle studies of the three films after 5 min showed that both SF-*g*-(AAc-*co*-EGDMA) (GY ~9.5%) and SF-*g*-(AAc-*co*-EGDMA) + Ag (GY ~9.5%) increased the surface wettability against DI (Figure 2b). The contact angle decreases from 105° (SF), which means the surface is highly hydrophobic, to 95° in the modified films, confirming a hydrophilic AAc graft onto the surface, as we reported in our last work [48]. The angle did not change between films with and without silver; thus, allowing us to conclude that silver does not affect the water absorption. Although silver was expected to increase hydrophilicity because of the metal–oxygen interactions, since the measurement was made almost instantaneously (after 5 min, to avoid the evaporation of the drop) no changes in the hydrophilicity between the two films were found.

In the case of ^13^C-CPMAS-SS-NMR spectra (see Supplementary Material) the signal observed at 0 ppm is assigned to the pendant -CH_3_ chains of SF and the signal at 29.7 ppm corresponds to the methylene groups that were formed after the grafting reaction [53]. The rest of the signals correspond to the AAc:EGDMA graft. The carbonyl group of acrylic and methacrylic compounds showed a chemical shift at 176.0 ppm, which is coherent with reports from the literature [54]. It is not possible to differentiate the carbonyl from AAc and that from dimethacrylate since they present structural similarities. In addition, at 19.9 and 62.0 ppm, the signal corresponding to methyl and methylene groups of EGDMA are observed. The signals at 44.6 and 38.5 ppm were assigned to the α-carbons and the carbonyl groups in AAc and EGDMA, respectively.

The effect of silver nitrate concentration on the grafting reaction was also evaluated. The simultaneous reaction was performed in the same conditions but altering the concentration of the salt. As in the first experiments, the synthesized films at different concentrations of AgNO_3_ became colorful, starting from a concentration above 5 mM of silver salt the films became orange with different intensities, similar to the color of the aqueous dispersion of small spherical and quasi-spherical AgNPs [55,56]. For all the reactions, the AAc:EGDMA GY was between ~8% and ~9.5%. The increase in the concentration of the silver nitrate in the reaction did not affect the grafting yield in the films (check Figure 2c), the amount of AAc:EGDMA graft onto the surface is still the same, and EWC% and hydrophilicity tests also confirm this.

Reports claim that the reaction time in the synthesis of AgNPs plays an essential role in their shape and size and thus the time was fixed for all reactions; therefore, to obtain higher doses (input energy to the system), the intensity of the radiation (I = [kGy/h]) was varied instead of time to control this variable for the following experiments. Two different radiation intensities were tested, and when I was decreased from 10 to 5 kGy h^−1^, the color of the modified SF changed, confirming that irradiation dose affect the properties of the synthesized materials as well as time.

**Figure 3:**
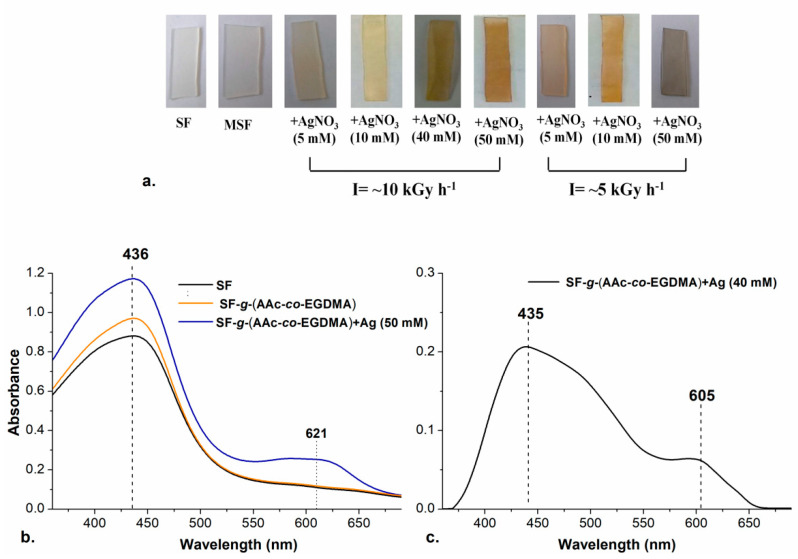
(**a**) Films prepared with different intensity of radiation and concentration of AgNO_3_; (**b**) UV-Vis spectra of (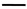) SF, (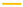) SF-*g*-(AAc-*co*-EGDMA) and (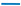) SF-*g*-(AAc-*co*-EGDMA) + Ag (50 mM); and (**c**) UV-Vis spectrum of SF-*g*-(AAc-*co*-EGDMA) + Ag (10 mM).

Three concentrations of AgNO_3_ were tested at I = ~5 kGy h^−1^, to check the effect of exposure to radiation in the formation of AgNPs with different shapes or sizes. Concentrations of 5, 10, and 50 mM of silver nitrate were evaluated. After exposure, SF developed different colors depending on the intensity of radiation (see Figure 3a). The typical coloration for aqueous colloids with spherical AgNPs with diameter <20 nm is usually yellow, while for particles bigger than 100 nm the coloration is most often blue [30,41,57]. The formation of bigger particles at 50 mM and lower intensity of radiation can be attributed to the increase in the time of exposure which promotes effective silver reduction and formation of bigger aggregates, according to Abedini et al., 2016.

Silver nanoparticles have a surface plasmon resonance (SPR) depending on the shape and size of the particle which can be detected by UV-Visible Spectroscopic analysis. The presence of the nanoparticles was confirmed in the different films by that technique. The spectra in Figure 3b,c show the typical bands for SRP of AgNPs.

The spherical nanoparticles yielded a SPR peak at 435 nm, similar to the value previously reported for spherical and quasi-spherical AgNPs with sizes below 20 nm [30]. It is noticeable that an additional band in 605 nm appeared, which is typical for triangular silver nanoplates or quasi-spherical AgNPs with a bigger size (~100 nm). The formation of the bigger particles occurred in the experiments with a higher concentration of AgNO_3_ and increased radiation exposure (I = ~5 kGy) This can be explained with the continuous reduction reactions happening while the radiation where silver clusters are easier to form.

**Figure 4:**
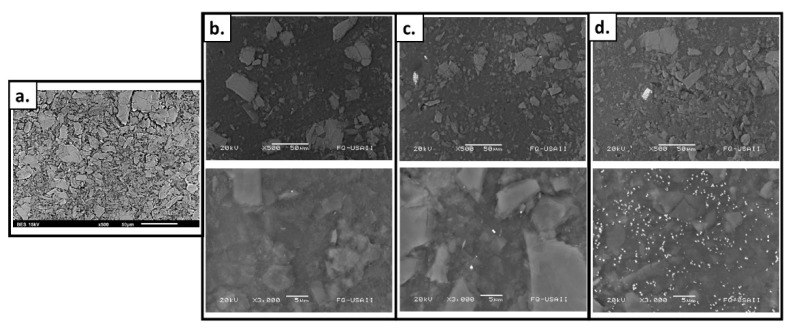
SEM images of (**a**) SF; (**b**) SF-*g*-(AAc-*co*-EGDMA); (**c**) SF-*g*-(AAc-*co*-EGDMA) + Ag (10 mM); and (**d**) SF-*g*-(AAc-*co*-EGDMA) + Ag (50 mM).

Due to the thickness of the films, images from transmission microscopy could not be obtained; however, scanning electron microscopy (SEM) was successful. The obtained micrographies show the morphologies of the films’ surfaces before (Figure 4a) and after the modification processes. The roughness of the films does not increase substantially, which was expected since the grafting percentage was low (max GY ~9.5%) (Figure 4b). However, the presence of silver particles is evident when looking at the images corresponding to the silver-functionalized materials. As expected, the amount of silver that can be observed depends on the concentration of silver used in the synthesis of the material (Figure 4c,d). Changes in the color of the films may be attributed to these silver aggregates or clusters inside or outside of the pores of the graft [58].

**Figure 5:**
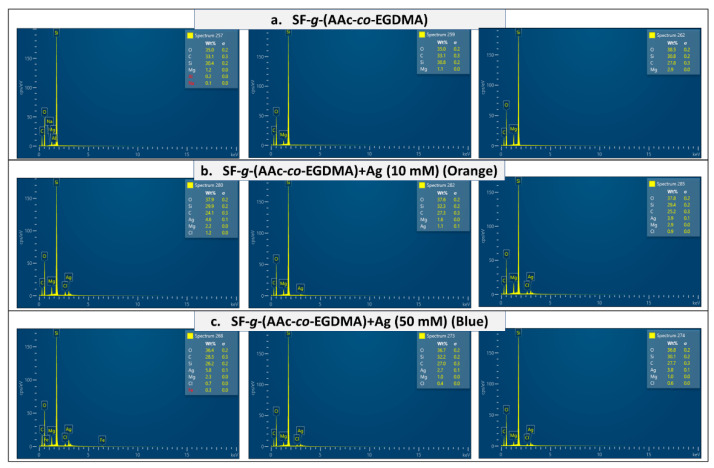
EDX spectra of (**a**) SF-*g*-(AAc-*co*-EGDMA), (**b**) SF-*g*-(AAc-*co*-EGDMA) + Ag (10 mM), and (**c**) SF-*g*-(AAc-*co*-EGDMA) + Ag (50 mM).

Additionally, the energy-dispersive X-ray spectrometry (EDX) information confirmed the presence of silver and the AAc:EDGMA graft on the surface. Films with a lower concentration of silver (Figure 5b) showed correspondingly lower amounts of silver. The section of the surface with a higher amount of silver reached 4.6%. In contrast, the blue film, synthesized using 50 mM of silver nitrate showed higher concentrations of silver in almost all the surface, the higher amount was nearly 6% (Figure 5c). For the film without silver, no amount of the metal was found in any spectra (Figure 5a). The elemental distributions for the films revealed a silver content that may be adequate concerning the development of antimicrobial surfaces [59].

To test for the stability of the materials, the films were exposed to atmospheric oxygen and water for 12 months and monitored periodically. Both tests were performed in the absence of light. The films remained without color changes for 12 months, suggesting the absence of oxidation reactions which would have darkened the samples. FTIR-ATR and UV-Vis spectra were recorded after 6 months and very similar spectral data was obtained (see Supplementary Material). In an aqueous solution, the diffusion of silver outside the polymer to the solution was not detected by absorbance measurements, this supports the fact that the particles and aggregates are occluded into the AAc grafts may be by a carboxylate-AgNP interaction in DI water and buffer solutions.

### 2.3. Antimicrobial Test

**Figure 6:**
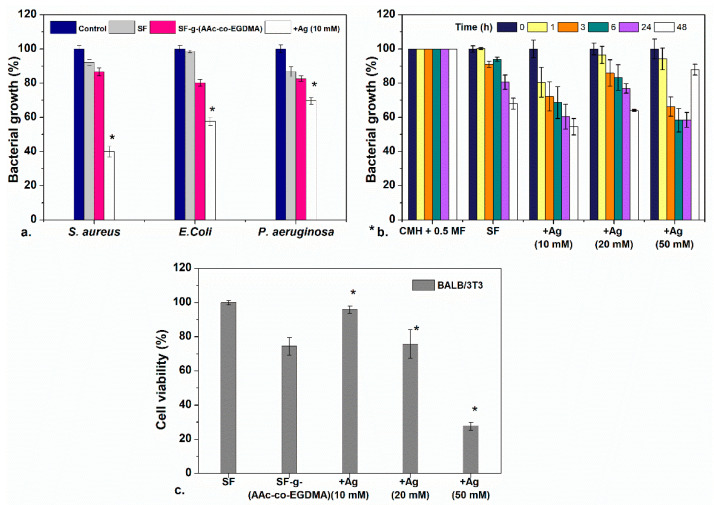
(**a**) Antimicrobial evaluation against three bacteria of SF, SF-*g*-(AAc-*co*-EGDMA), and SF-*g*-(AAc-*co*-EGDMA) + Ag. (**b**) Antimicrobial effect of SF-*g*-(AAc-*co*-EGDMA) + Ag with different concentrations of Ag as a function of the time against *S. aureus*, and (**c**) cytocompatibility of the films in fibroblast cultures after 24 h. Statistically significant differences between control samples and modified materials are highlighted with a *.

The film: SF-*g*-(AAc-*co*-EGDMA) + Ag (10 mM) (orange) was used for preliminary antimicrobial analyses against Gram-positive and Gram-negative bacteria. The results were compared between this sample, the film without silver and pristine silicone. As seen in Figure 6a, UV–Vis results confirmed growth inhibition due to the presence of SF-*g*-(AAc-*co*-EGDMA) + Ag (10 mM) for the three bacteria after 24 h according to absorbance measurements.

According to these results, SF-*g*-(AAc-*co*-EGDMA) + Ag (10 mM) showed better inhibition for *S. aureus* in which the bacterial growth decreases up to a value of ~40% of cells (in comparison to 100% for other samples) after 24 h. For *E. coli*, the inhibition was less significant, as well as for *P. aeruginosa,* the inhibiting effect was less dramatic; however, slight growth inhibition is still observed, which is common for silver modified materials. To examine if these differences are significant, a one-way ANOVA difference of means test with a confidence level of *p* = 0.01 was performed (see Section 3 and Supplementary Material) between the culture media containing a pristine SF, and assays involving either the modified films.

One accepted mechanism for the antimicrobial effect of the AgNPs is that they attach to the cell wall of bacteria and disrupt the biochemical processes of the cell. The nanoparticles can pass through the cell wall and affect the environment inside the bacterium triggering cell death [60]. The difference between the thickness in the cell wall is an aspect that directly influences the bactericidal effect of the particles when those penetrate the membrane and get inside the cell; nevertheless, this antimicrobial effect can be reached only by contact too as it has been previously demonstrated by microscopy [61]. The linkage between the metallic particle and the cell wall can be conducted by electrostatic forces between these two entities, but this depends on the coating of the particle. For instance, particles coated with polycarboxylates are normally negatively charged [62,63]; therefore, these particles may interact with the proteins in the cell wall causing irreversible changes in the cell wall structure and cell morphology; these changes conduct to cell death [20,64,65].

Figure 6a shows inhibition for both Gram-negative (*E. coli* and *P. aeruginosa*) and Gram-positive *S. aureus*. Despite what previous reports suggest, the antibacterial effect was dramatic for *S. aureus* than *E. coli* and *P. aeruginosa*, even though *S. aureus* is a Gram-positive bacteria. The higher bactericidal effect against *S. aureus* can be attributed to the antimicrobial mechanism is by direct contact between the cell wall and the AgNP surface, as it has been reported for Gram-positive *S. epidermidis* in glass surfaces modified with AgNPs which showed antibacterial effect by cell/surface contact [66,67]. For Gram-negative bacteria the main antimicrobial mechanism is attributed to the release of silver ions Ag^+^ produced by oxidation of AgNPs [68]. Something beyond the scope of this paper which is interesting to remark is that although inhibition of Gram-positive bacteria due to the presence of AgNPs is not unprecedented, the behavior seen on this work unexpected for these materials and it would be interesting to investigate further in follow-up investigations.

After demonstrating that the bactericidal activity was more effective for *S. aureus*, the performance of the SF prepared with different concentrations of silver was tested. The bacterial growth inhibition was evaluated as a function of time (Figure 6b). This graph shows that the bacterial growth of *S. aureus* is inhibited for up to 48 h when testing the lower silver concentration films. The three tested films showed bactericidal effect after 24 and 48 h. There are no differences in the results between the films with lower and higher concentration of silver.

As it may be noted, the inhibition of the film prepared with a concentration of 50 mM AgNO_3_ has an apparent anomalous increase in bacterial growth at 48 h. This can be explained as an interference effect of the oxidation of AgNPs and aggregation of silver oxide in the presence of light and oxygen, which increase the absorbance of the medium (silver oxides are black insoluble solids) and could be confused with bacterial growth on absorbance measurements. This effect is not seen in materials with lower concentrations of silver because the oxidation and aggregation are more probable in materials with more concentrations of silver.

The antibacterial effect of the AgNPs depends on the particle size; small-diameter particles have a larger area and thus more contact with the bacteria. For that reason, the films prepared with 10 mM of silver nitrate with an excess of small spherical particles showed the same effect as the films containing more Ag (50 mM), because the effect is due to the size of the nanoparticles rather than to the concentration.

### 2.4. Cytocompatibility

Cell viability was evaluated in small volumes of growth medium in direct contact with BALB/3T3 (mouse). As shown in Figure 5c, cell viability in this cell line was unaffected for the films with a low concentration of silver (10 mM), for these samples, good cytocompatibility (>90%) was observed at 24 h. It is important to remark that the cell viability of that film is better than that of film modified only with the AAc:EGDMA grafts. All the evaluated films had a GY ~10%, and it is shown that with an increase in the GY onto the silicone surface, the cytocompatibility decreases considerably [69]. An increase in Ag concentration directly affects the cell viability due to the cytotoxicity of the nanoparticles. The film modified with 50 mM of silver nitrate induces cell death, which can be seen since the cell viability decreased below 40%. Fibroblasts are adherent cells; thus, they need to be attached to a surface to function, therefore the fact that they survived in solution is a good sign. In comparison to other studies in which lower cytocompatibilities have been found, the cytocompatibility of these materials is acceptable [70].

## 3. Experimental Section

### 3.1. Materials and Methods

Silicone films (1 × 4 cm) with a density ranging from 1.1 to 1.5 g cm^−3^ and a thickness of 1 mm were purchased from Good-fellow (Huntingdon, UK). Silver nitrate (99.9%), ethylene glycol dimethacrylate, and acrylic acid were acquired from Sigma-Aldrich Co. (St. Louis, MO, USA). All the monomers were purified under vacuum distillation before used. Deionized water and ethanol (analytical grade) were obtained from Baker Mexico and were used without further purification.

### 3.2. Grafting Reaction and Silver Reduction Assisted by Radiation

As preliminary experiments, silicone films (SF) were introduced in an ampoule containing 8 mL of an AAc:EGDMA mixture in 3:1 and 5:1 ratios (SF-*g*-(AAc-*co*-EGDMA)). The monomer concentrations tested were 10 and 20% (*v*/*v*) in EtOH:H_2_O (1:1) mixture as a solvent.

In silicone films with silver immobilization (SF-*g*-(AAc-*co*-EGDMA) + Ag), different solutions with a concentration of silver nitrate were used (1, 5, 10, 20, 30, 40, and 50 mM) the AgNO_3_ was dissolved in the monomer/EtOH:H_2_O solutions. Monomer concentration was fixed at 20% *v/v* in a ratio 3:1 (AAc:EGDMA). The ampoules were deoxygenated by displacing oxygen with Argon for 20 min subsequently the ampoules were sealed and later exposed to variable doses of ^60^Co γ-radiation (I = ~5 and ~10 kGy h^−1^) using a Gammabeam 651PT available in the Institute of Nuclear Sciences (ICN) at the National Autonomous University of Mexico (UNAM) to initiate the polymerization reaction. After exposing the films to radiation, all the modified SF were washed in different solvents to remove the occluded solvent, AgNO_3_, monomer, and copolymer residues. Finally, the samples were dried under vacuum (−80 kPa) at 40 °C and weighed.

The grafting yield (GY) was calculated from the weight difference between pristine PDMS (*m*_0_) and grafted-PDMS (*m_g_*), the general formula is shown in Equation (1):

GY (%) = [(*m_g_* − *m*_0_)/*m*_0_]100 (1)

All the experiments were repeated thrice, and the standard error of the mean (Err) was calculated for all measurements.

### 3.3. Characterization

To check for a successful modification of the materials the Fourier Transform Infrared Spectroscopy Attenuated Total Reflectance Spectroscopy (FTIR ATR) using a Perkin-Elmer Spectrum 100 spectrometer (Perkin Elmer Cetus Instruments, Norwalk, CT, USA). All the materials were dried under a vacuum before the measurements. The characterization of the surfaces was made using UV-VIS spectroscopy using an Ocean Optics HR4000CG-UV-NIR, and the Scanning Electron Microscopy (SEM) in a JEOL JSM 5900 LV with graphite recovering at 20 kV and energy-dispersive X-ray spectrometry (EDX). Since both FTIR ATR measurements are performed on the solid state, sample preparation only consisted of the aforementioned vacuum dry.

The equilibrium water content (EWC%) was measured in deionized water for 48 h each, the excess of water was removed using filter paper and then the films were weighted. The swelling degree (SD) was calculated using Equation (2):

SD (%) = [(*m_S_* − *m_g_*)/*m_g_*]100 (2)

*m_g_* and *m_s_* represent the mass of the grafted and the swollen film, respectively. All the experiments were repeated thrice, and the standard error of the mean (Err) was calculated for all measurements.

For contact angle a Kruss DSA 100 drop shape analyzer (Matthews, NC, USA) was used, the angle was recorded at 5 min after deionized water droplet had been deposited into the dry samples. All the experiments were made thrice.

### 3.4. Bacterial-Growth Inhibition Tests

Small pieces of SF, SF-*g*-(AAc-*co*-EGDMA), SF-*g*-(AAc-*co*-EGDMA) + Ag (1 cm^2^ and 100 mg), were placed in tubes containing *Staphylococcus aureus* (ATCC 25,923), *Escherichia coli* (ATCC 25,922), or *Pseudomonas aeruginosa* (ATCC 27,853) in Muller–Hinton agar at a concentration of approximately 1.5 × 10^8^ CFU mL^−1^ (0.5 McFarland) and then incubated at 37 °C for 3, 6, 12, 24, and 48 h. Control cultures with and without bacteria were also prepared with this method. To quantify bacterial growth, light absorption by the culture media was measured in a UV–Vis spectrophotometer at wavelengths ranging from 450 to 800 nm. Absorbance at 600 nm was used to compare bacterial growth in all the systems as stated in the literature [71]. All bacterial-growth inhibition tests were performed three times and a one-way ANOVA difference of means test (*p* = 0.01) was conducted to evaluate the statistical significance of the results at 24 h. The details of the ANOVA test are presented in the Supplementary Material. For these assays, since the means were expected to be different if growth inhibition was achieved; therefore, these were the proposed hypotheses for the statistical test:

H_0_: % Growth_SF_ = % Growth_SF-g-(AAc-*co-*EDGMA)_ = % Growth_SF-g-(AAc-*co-*EDGMA) + Ag_

H_a_: % Growth_SF_ ≠ % Growth_SF-g-(AAc-*co-*EDGMA)_ ≠ % Growth_SF-g-(AAc-*co-*EDGMA) + Ag_

Post-hoc (Tukey test) tests allowed to determine which of the samples had indeed different ability to stop growth inhibition for each bacteria.

### 3.5. Cytocompatibility Essays

The cytocompatibility tests were performed using a murine embryonic fibroblast cell line BALB/3T3 (ATCC CCL-163, Manassas, VA, USA). The fibroblast cell line was cultured in Dulbecco’s modified Eagle’s medium (DMEM) with FBS 10% (fetal bovine serum), penicillin-streptomycin (1% *w*/*v*), and gentamicin (10 µg/mL). Experiments were performed in 96 well plates with 50,000 cells mL^−1^ for 12 h incubated in a humidified atmosphere of 5% CO2 at 37 °C. SF-g-(AAc-co-EGDMA), and SF-g-(AAc-co-EGDMA) + Ag films (0.25 × 0.20 cm), were submerged in the cell media for 24 h in culture standard conditions. After this time, the films were removed and cell viability was determined using a MTT kit (Roche, Switzerland). All experiments were performed thrice and compared with cells without films as a negative control. Finally, absorbances were obtained using a Multiskan FC, Thermo Scientific spectrophotometer at 620 nm. Cytocompatibility (%) is calculated as follows:

Cytocompatibility (%) = [A_sample_/A_cont_]100 (3)

All cytocompatibility tests were performed three times and a one-way ANOVA difference of means test (*p* = 0.01) was conducted to evaluate the statistical significance of the results at 24 h. The details of the ANOVA test are presented in the Supplementary Material. For these assays, since the means were expected to be different if growth inhibition was achieved; therefore, these were the proposed hypotheses for the statistical test:

H_0_: % Cell viability_SF_ = % Cell viability_SF-g-(AAc-*co-*EDGMA)_ = % Cell viability_SF-g-(AAc-*co-*EDGMA) + Ag (10 mM)_ = % Cell viability_SF-g-(AAc-*co-*EDGMA) + Ag (20 mM)_ = % Cell viability_SF-g-(AAc-*co-*EDGMA) + Ag (50 mM)_.

H_a_: % Cell viability_SF_ ≠ % Cell viability_SF-g-(AAc-*co-*EDGMA)_ ≠ % Cell viability_SF-g-(AAc-*co-*EDGMA) + Ag (10 mM)_ ≠ % Cell viability_SF-g-(AAc-*co-*EDGMA) + Ag (20 mM)_ ≠ % Cell viability_SF-g-(AAc-*co-*EDGMA) + Ag (50 mM)_ post-hoc (Tukey test) tests allowed to determine which of the samples had differences in their cytocompatibility.

## 4. Conclusions

The simultaneous polymer grafting and silver reduction using gamma radiation were successfully reached in ethanol:water solution. With the analysis of the obtained materials, it was possible to determine the necessary conditions to obtain reproducible grafts while also determining the synthetic conditions that allow for AgNPs to form within the polymer grafts. The presence of silver on the SF was verified by EDX elemental analysis, UV-vis determination of plasmon resonance, and SEM microscopy. As suggested through the change of color of the films from clear to orange, the formation of spherical or quasi-spherical AgNPs.

The modified films SF-*g*-(AAc-*co*-EGDMA) + Ag showed good antimicrobial activity against *S. aureus* and some antimicrobial activity of *E. coli* and *P. aeruginosa* while maintaining and a cytocompatibility (above 90%) as long as the concentration of silver was 10 mM or lower.

## Data Availability

Data sharing is not applicable to this article.

## Supplementary Materials

The following are available online, Figure S1: EWC% for the films in DI water and PBS; Figure S2: ^13^C CPMAS SS NMR spectra of the different films; Figure S3: FTIR-ATR spectrum of the SF-*g*-(AAc-*co*-EGDMA) + Ag (50 mM) recorded after 12 months; Figure S4: UV-Vis spectrum of the SF-*g*-(AAc-*co*-EGDMA) + Ag (50 mM) recorded after 12 months.

## Sample Availability

Samples of the compounds are not available from the authors.
