# Supplementary material for: Simultaneous Grafting Polymerization of Acrylic Acid and Silver Aggregates Formation by Direct Reduction Using γ Radiation onto Silicone Surface and Their Antimicrobial Activity and Biocompatibility
Source: Molecules. 2021 May 12;26(10):2859. doi: 10.3390/molecules26102859 (PMC8151000; doi:10.3390/molecules26102859)
Supplement: Supplementary file 1 [file molecules-26-02859-s001.zip › molecules-1203693-supplementary.pdf]

Supplementary material for:

Simultaneous grafting polymerization of acrylic acid and silver aggregates formation by direct reduction using g radiation onto silicone surface and their antimicrobial activity and biocompatibility

Marlene A. Velazco-Medel[1*], Luis A. Camacho-Cruz[1], Héctor Magaña[2], Kenia Palomino[2] and Emilio Bucio[1*]

*Correspondence: ebucio@nucleares.unam.mx (E.B.C.); marlene.velazco@correo.nucleares.unam.mx (M.A.V-M)

Figure S1: EWC% for the films in DI water and PBS

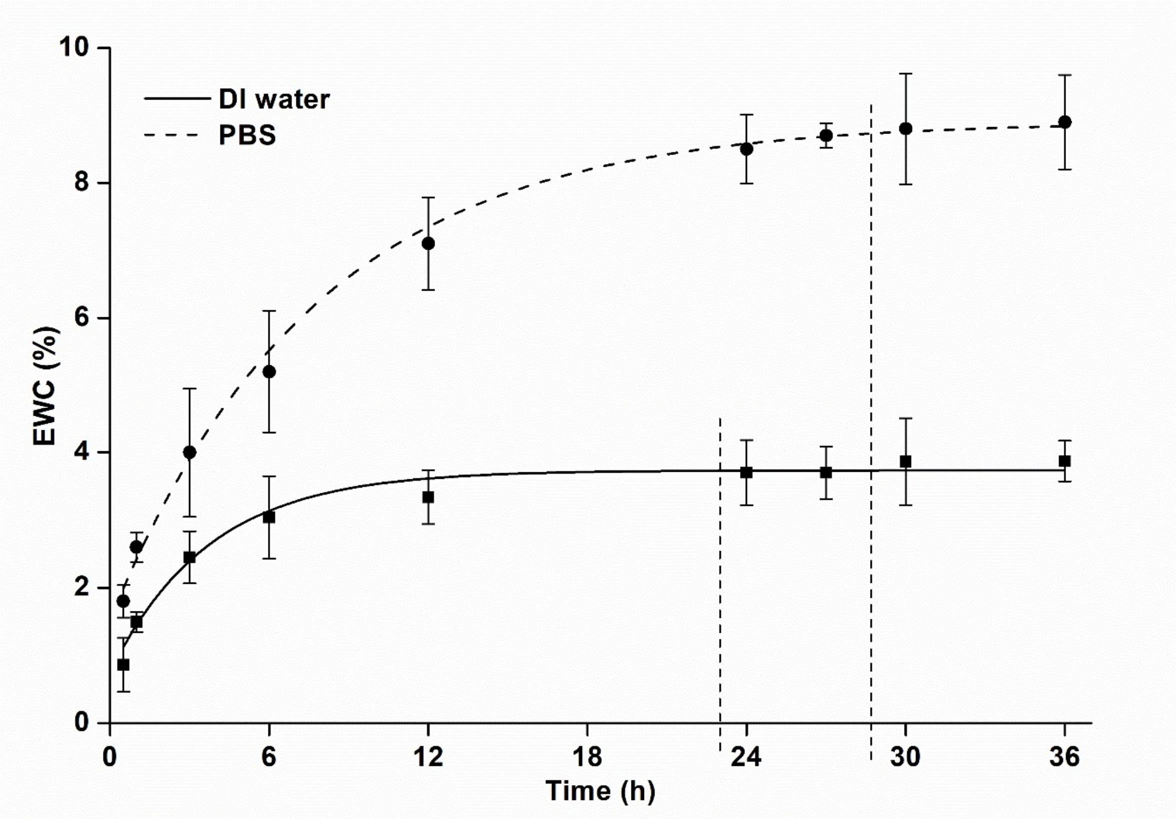

Figure S2: ^{13}C CPMAS SS NMR spectra of the different films.

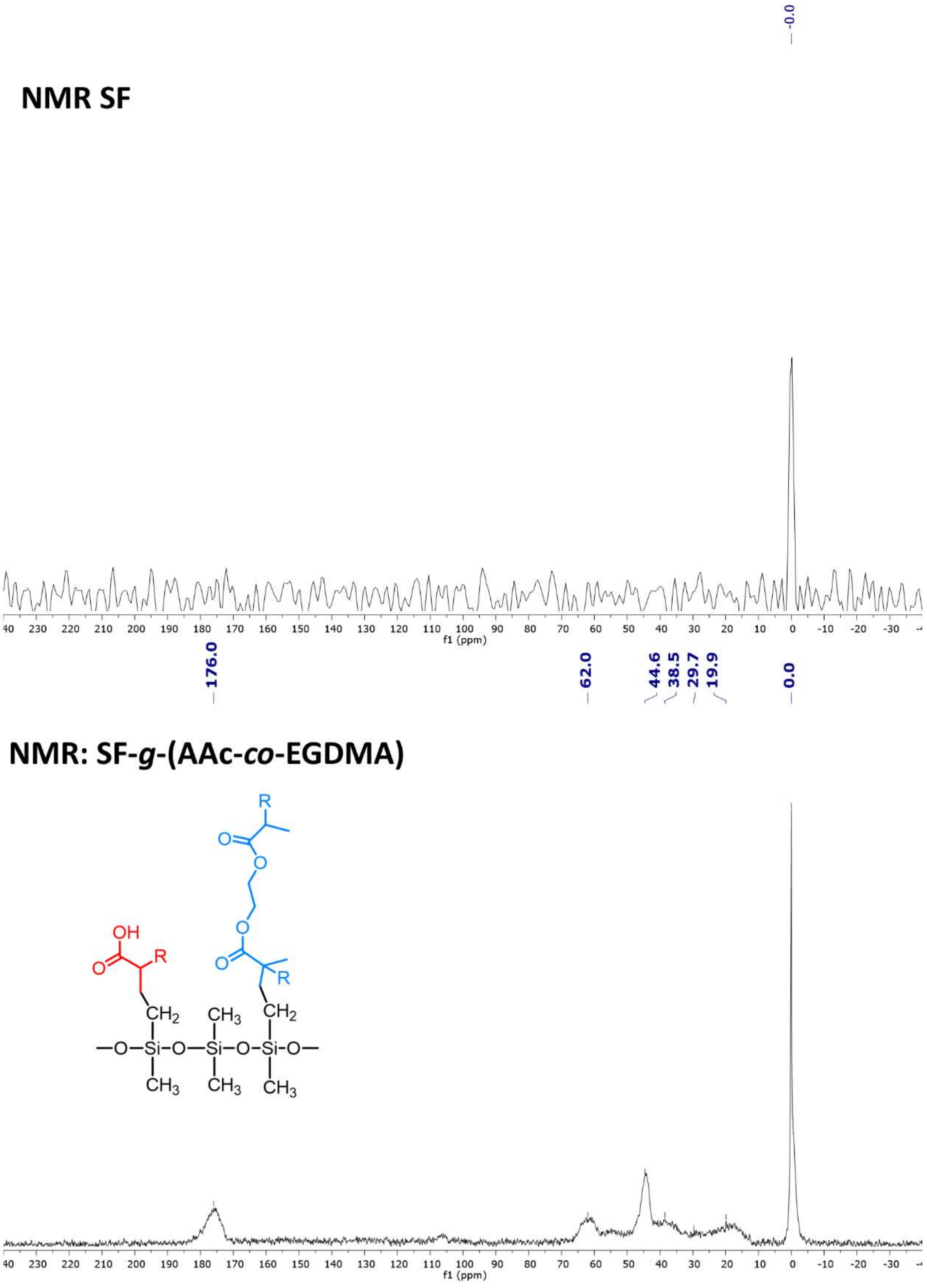

Figure S3: FTIR-ATR spectrum of the SF-*g*-(AAc-*co*-EGDMA)+Ag (50 mM) recorded after twelve months

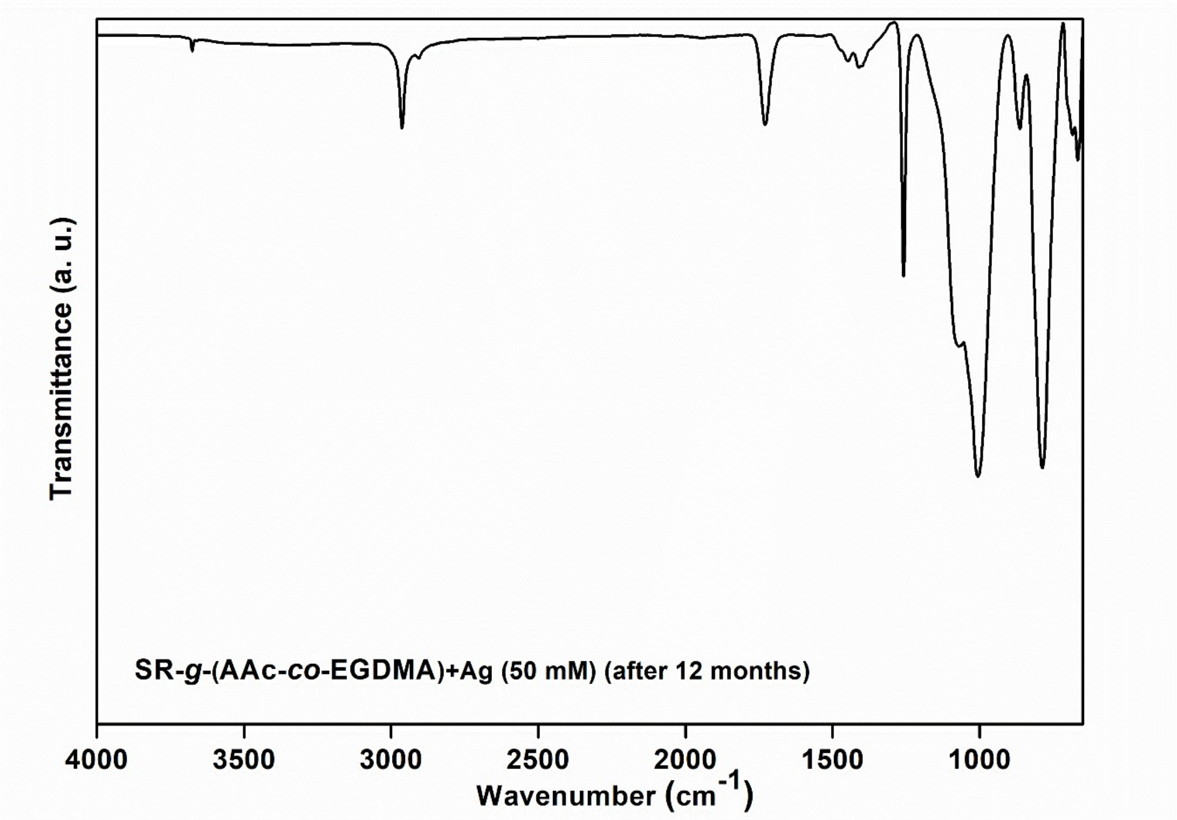

Figure S4: UV-Vis spectrum of the SF-*g*-(AAc-*co*-EGDMA)+Ag (50 mM) recorded after twelve months

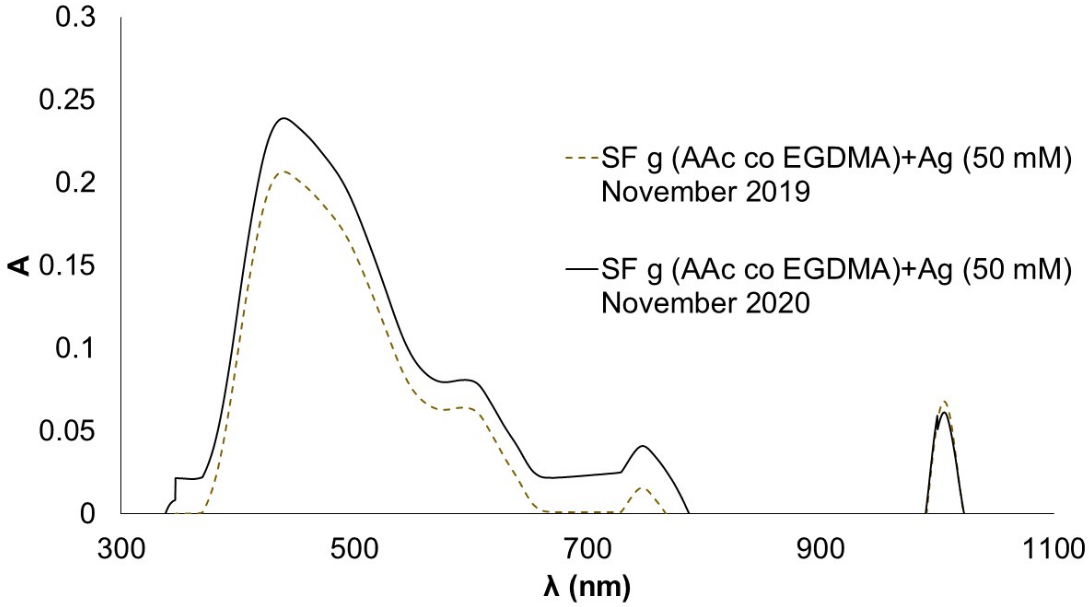

One-Way ANOVA difference of means hypothesis test with post-hoc test (Tukey)

S. aureus **growth inhibition**

H_0: % $Growth_{SF}$ = % $Growth_{SF\text{-}g\text{-}(AAc\text{-}co\text{-}EDGMA)}$ = % $Growth_{SF\text{-}g\text{-}(AAc\text{-}co\text{-}EDGMA)+Ag}$
H_a: % $Growth_{SF}$ ≠ % $Growth_{SF\text{-}g\text{-}(AAc\text{-}co\text{-}EDGMA)}$ ≠ % $Growth_{SF\text{-}g\text{-}(AAc\text{-}co\text{-}EDGMA)+Ag}$

**ANOVA**			
**Sample**	SF	SF-g-(AAc-*co*-EDGMA)	SF-g-(AAc-*co*-EDGMA)+Ag
**Repetition 1**	92.9	89.6	34.9
**Repetition 2**	88.8	82.1	39.2
**Repetition 3**	94.8	85.7	45.4
**Mean**	92.166	85.800	39.833
**St. Deviation**	3.066	3.651	5.278

**Source**	**Sum of squares**	**Degrees of Freedom**	**Mean Square**	**F-value**	**p-value**
**Sample**	4892.2467	2	2446.1233	142.9460	8.6854×10^{-6}
**Error**	102.6733	6	17.1122		
**Total**	4994.9200	8			

Null hypothesis is **rejected (p<0.01)**. Therefore, there is a significant difference between the three samples

**Post-Hoc**			
**Sample pair**	**Tukey HSD Q statistic**	**Tukey HSD p-value**	**Tukey HSD interfence**
SF and SF-g-(AAc-*co*-EDGMA)	2.6658	0.22255	Insignificant (p≥0.05)
SF and SF-g-(AAc-*co*-EDGMA) + Ag	21.9122	0.0010053	Significant (p<0.01)
SF-g-(AAc-*co*-EDGMA) and SF-g-(AAc-*co*-EDGMA)+ Ag	19.2464	0.0010053	Significant (p<0.01)

***E. coli* growth inhibition**

H_0: % Growth$_{SF}$ = % Growth$_{SF\text{-}g\text{-}(AAc\text{-}co\text{-}EDGMA)}$ = % Growth$_{SF\text{-}g\text{-}(AAc\text{-}co\text{-}EDGMA)+Ag}$
H_a: % Growth$_{SF}$ ≠ % Growth$_{SF\text{-}g\text{-}(AAc\text{-}co\text{-}EDGMA)}$ ≠ % Growth$_{SF\text{-}g\text{-}(AAc\text{-}co\text{-}EDGMA)+Ag}$

ANOVA

Sample	SF	SF-g-(AAc-*co*-EDGMA)	SF-g-(AAc-*co*-EDGMA)+Ag
**Repetition 1**	97.1	75.6	53.4
**Repetition 2**	99.7	82.1	56.9
**Repetition 3**	98.7	80.9	61.5
**Mean**	98.500	79.533	57.2667
**St. Deviation**	1.312	3.459	4.062

Source	Sum of squares	Degrees of Freedom	Mean Square	F-value	p-value
**Sample**	2555.7267	2	1277.8633	126.9961	1.2291×10^{-5}
**Error**	60.3733	6	10.0622		
**Total**	2616.1000	8			

Null hypothesis is **rejected (p<0.01)**. Therefore, there is a significant difference between the three samples

Post-Hoc

Sample pair	Tukey HSD Q statistic	Tukey HSD p-value	Tukey HSD interfence
SF and SF-g-(AAc-*co*-EDGMA)	10.3563	0.0010053	Significant (p<0.01)
SF and SF-g-(AAc-*co*-EDGMA) + Ag	22.5145	0.0010053	Significant (p<0.01)
SF-g-(AAc-*co*-EDGMA) and SF-g-(AAc-*co*-EDGMA)+ Ag	12.1582	0.0010053	Significant (p<0.01)

***P. aeruginosa* growth inhibition**

H_0: % $Growth_{SF}$ = % $Growth_{SF\text{-}g\text{-}(AAc\text{-}co\text{-}EDGMA)}$ = % $Growth_{SF\text{-}g\text{-}(AAc\text{-}co\text{-}EDGMA)+Ag}$

H_a: % $Growth_{SF}$ ≠ % $Growth_{SF\text{-}g\text{-}(AAc\text{-}co\text{-}EDGMA)}$ ≠ % $Growth_{SF\text{-}g\text{-}(AAc\text{-}co\text{-}EDGMA)+Ag}$

ANOVA

Sample	SF	SF-g-(AAc-*co*-EDGMA)	SF-g-(AAc-*co*-EDGMA)+Ag
**Repetition 1**	92.6	79.6	73.5
**Repetition 2**	83.1	82.8	66.9
**Repetition 3**	85.7	84.9	68.5
**Mean**	87.133	82.433	69.633
**St. Deviation**	4.909	2.668	3.442

Source	Sum of squares	Degrees of Freedom	Mean Square	F-value	p-value
**Sample**	492.1800	2	246.0900	17.1372	0.0033
**Error**	86.1600	6	14.3600		
**Total**	578.3400	8			

Null hypothesis is **rejected (p<0.01)**. Therefore, there is a significant difference between the three samples

Post-Hoc

Sample pair	Tukey HSD Q statistic	Tukey HSD p-value	Tukey HSD interfence
SF and SF-g-(AAc-*co*-EDGMA)	2.1482	0.3476896	Insignificant (p≥0.05)
SF and SF-g-(AAc-*co*-EDGMA) + Ag	7.9987	0.0031531	Significant (p<0.01)
SF-g-(AAc-*co*-EDGMA) and SF-g-(AAc-*co*-EDGMA)+ Ag	5.8505	0.0143312	Significant (p<0.05)

Cytocompatibility

H_0: % Cell viability$_{SF}$ = % Cell viability$_{SF\text{-g-(AAc-}co\text{-EDGMA)}}$ = % Cell viability$_{SF\text{-g-(AAc-}co\text{-EDGMA)+Ag (10 mM)}}$ = % Cell viability$_{SF\text{-g-(AAc-}co\text{-EDGMA)+Ag (20 mM)}}$ = % Cell viability$_{SF\text{-g-(AAc-}co\text{-EDGMA)+Ag (50 mM)}}$
H_a: % Cell viability$_{SF}$ ≠ % Cell viability$_{SF\text{-g-(AAc-}co\text{-EDGMA)}}$ ≠ % Cell viability$_{SF\text{-g-(AAc-}co\text{-EDGMA)+Ag (10 mM)}}$ ≠ % Cell viability$_{SF\text{-g-(AAc-}co\text{-EDGMA)+Ag (20 mM)}}$ ≠ % Cell viability$_{SF\text{-g-(AAc-}co\text{-EDGMA)+Ag (50 mM)}}$

ANOVA

**Sample**	Blank (A)	SF (B)	SF-g-(AAc-*co*-EDGMA) (C)	SF-g-(AAc-*co*-EDGMA)+Ag (10 mM) (D)	SF-g-(AAc-*co*-EDGMA)+Ag (20 mM) (E)	SF-g-(AAc-*co*-EDGMA)+Ag (50 mM) (F)
**Repetition 1**	0.482	0.458	0.406	0.447	0.395	0.136
**Repetition 2**	0.491	0.479	0.323	0.435	0.409	0.107
**Repetition 3**	0.468	0.478	0.324	0.474	0.267	0.149
**Mean**	0.4803	0.4717	0.3510	0.4520	0.3570	0.1307
**St. Deviation**	0.0116	0.0118	0.0476	0.0200	0.0783	0.0215

**Source**	**Sum of squares**	**Degrees of Freedom**	**Mean Square**	**F-value**	**p-value**
reatment	0.2609	5	0.0522	32.8524	1.3342×10^{-6}
error	0.0191	12	0.0016		
total	0.2799	17			

Null hypothesis is **rejected (p<0.01)**. Therefore, there is a significant difference between the three samples

Post-Hoc

**Sample pair**	**Tukey HSD Q statistic**	**Tukey HSD p-value**	**Tukey HSD interfence**
A vs B	0.3767	0.899995	Insignificant (p≥0.05)
A vs C	5.6211	0.017711	Significant (p<0.05)
A vs D	1.2314	0.899995	Insignificant (p≥0.05)
A vs E	5.3603	0.0242	Significant (p<0.05)
A vs F	15.1973	0.0010	Significant (p<0.01)
B vs C	5.2444	0.027777	Significant (p<0.05)
B vs D	0.8548	0.899995	Insignificant (p≥0.05)
B vs E	4.9837	0.037922	Significant (p<0.05)
B vs F	14.8206	0.001005	Significant (p<0.01)
C vs D	4.3897	0.076532	Insignificant (p≥0.05)
C vs E	0.2608	0.899995	Insignificant (p≥0.05)
C vs F	9.5762	0.001005	Significant (p<0.01)
D vs E	4.1289	0.103262	Insignificant (p≥0.05)
